# Glycemic control with a basal-bolus insulin protocol in hospitalized diabetic patients treated with glucocorticoids: a retrospective cohort study

**DOI:** 10.1186/s12902-018-0300-0

**Published:** 2018-10-29

**Authors:** Elena Chertok Shacham, Hila Kfir, Naama Schwartz, Avraham Ishay

**Affiliations:** 1 Endocrinology Unit, Haemek Medical Center, Rabin Ave 21, Afula, Israel, 18134 Afula, Israel; 2 Statistics Department, Haemek Medical Center, Afula, Israel

**Keywords:** Type 2 diabetes mellitus, Basal-bolus insulin protocol, Glucocorticoid treatment, Glycemic control

## Abstract

**Background:**

Improved glycemic control is the desired outcome after the discharge of patients with diabetes. We aimed to determine the efficacy of a basal-bolus insulin protocol in hospitalized patients with diabetes treated with glucocorticoids.

**Methods:**

A retrospective cohort study compared the glycemic control of 150 hospitalized patients with diabetes and elevated inflammatory markers who were either treated with (*n* = 61) or without glucocorticoids (*n* = 89). All patients were treated with a basal-bolus regimen.

**Results:**

Glycosylated hemoglobin A1C (HbA1C) levels, mode of diabetes treatment before admission, length of hospitalization and inflammatory markers were similar in both groups of patients (treated and untreated with glucocorticoid). There was a trend toward female predominance in the glucocorticoid-treated group. Mean daily glucose levels were higher in patients taking glucocorticoids when compared with untreated patients (12.5 ± 2.7 mmol/l vs. 10.9 ± 2.4 mmol/l, *p* < .0001), and significantly higher at 5:00 PM (13.1 ± 3.4 vs. 10.2 ± 3 mmol/l, *p* < .0001), and 8:00 P.M. (13.9 ± 4.1 mmol/l vs. 11 ± 3.1 mmol/l, *p* < 0.001) . No difference was detected between the two groups in prandial and basal insulin doses during hospitalization. Overall, 64% of patients in the glucocorticoid-treated group versus 39% in the untreated group had inadequate glycemic control during hospitalization (*p* = 0.003).

**Conclusion:**

A significantly higher percentage of patients with diabetes who were treated with glucocorticoids during hospitalization did not achieve glycemic control with a basal-bolus insulin protocol. These patients had significantly higher mean blood glucose levels due to elevated levels in the afternoon and evening**.** New basal-bolus protocols with appropriate adjustments of short acting insulin are needed to treat patients with diabetes on glucocorticoid therapy.

## Background

The deleterious effects of hyperglycemia on the length of hospitalization, rate of infection, in-hospital mortality, and disability after hospitalization are well known from previous studies [1–3], so enhancing glycemic control during the hospital stay is a reasonable and logical target for improving patient outcomes [4, 5]. The management of patients with diabetes in noncritical care settings is based on basal-bolus insulin protocols according to contemporary guidelines [6, 7]. The recommended pre-meal target glucose levels for the majority of inpatients are 100–140 mg/dl (5.6–7.8 mmol/l), and random blood glucose levels should be less than 180 mg/dl (10.0 mmol/l) [7].

Glucocorticoids are widely used for treating inflammatory, autoimmune and malignant diseases [8]. New-onset hyperglycemia and difficulties in controlling existing diabetes are known to accompany glucocorticoid treatment [8, 9]. Indeed, a number of studies have reported elevated blood glucose levels in more than 50% of patients without diabetes who were treated with glucocorticoids during hospitalization [10–12].

Although a growing body of studies have examined various glycemic control protocols for glucocorticoid-treated patients with diabetes [13–15], there are still no uniform guidelines, as previous studies failed to demonstrate benefits of one regimen over another, and there is a paucity of randomized clinical trials. The aim of this study was to examine the efficacy of the currently implemented basal bolus insulin protocol in our institution among patients with diabetes who were hospitalized with an acute inflammatory state, and to examine whether there is a difference in glycemic control between those receiving glucocorticoids and those who were not.

## Methods

**Fig. 1:**
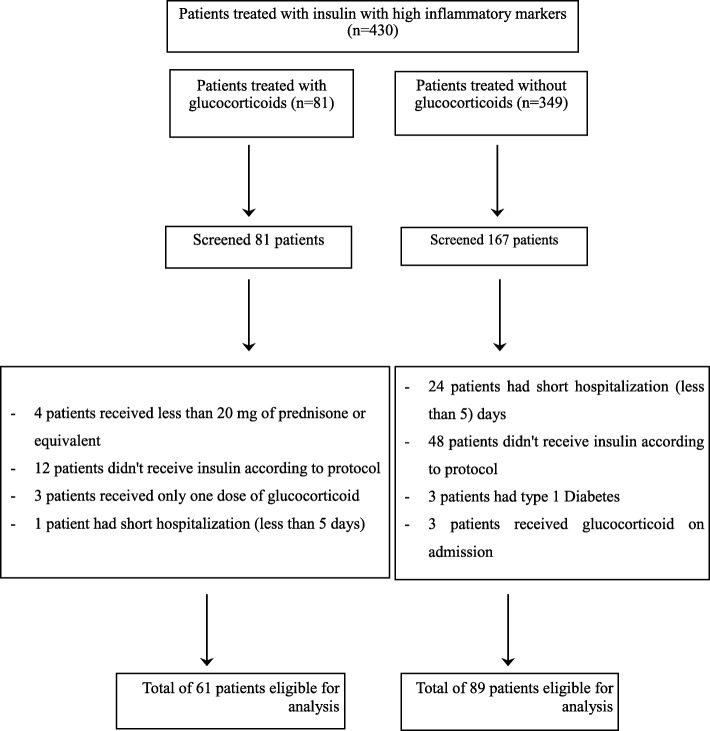
Flow chart showing the Screening and Eligibility process

This retrospective study was approved by HaEmek Medical Center Research Ethics Committee (Afula, Israel). We reviewed the electronic charts of patients diagnosed with type 2 diabetes mellitus (T2DM) admitted to the internal medicine department of the Medical Center from 2013 to 2015 and treated with a basal-bolus insulin protocol during their stay. Patients were included in the analysis if they were hospitalized for a minimum of 4 days, were 18 years of age or older, and had elevated inflammatory markers (C-reactive protein [CRP] ≥20) on admission. Exclusion criteria were pregnancy, type 1 diabetes and the presence of diabetic ketoacidosis. A total of 150 patients met the inclusion criteria: 61 who received glucocorticoid treatment during hospitalization (with prednisone ≥10 mg/day or an equivalent dose of glucocorticoid) and 89 who did not (Fig. 1).

The amounts of long- and short-acting insulin were recorded in units/day. The baseline characteristics collected at admission were recorded for each group. The most recent glycosylated hemoglobin A1C (HbA1C) for each patient (in the preceding 3 months) was retrieved.

**Fig. 2:**
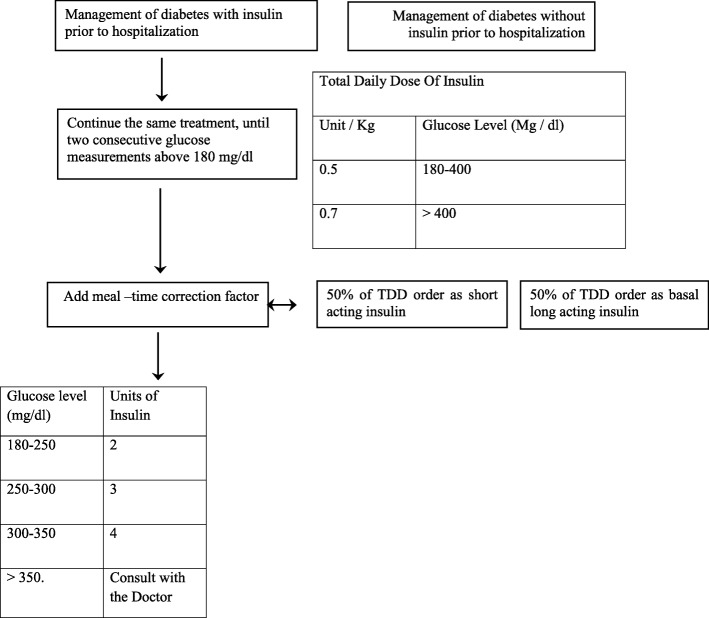
Hospital basal-bolus insulin protocol at admission

Both patient groups were treated according to the hospital’s standard protocol for treating of patients with hyperglycemia. The protocol was written by an endocrinologist, a clinical pharmacologist and a nurse responsible for diabetes treatment in our hospital. The treatment protocol was first implemented in July 2011 and has been used since then as the standard recommended care for diabetes in the hospital’s internal medicine departments (Fig. 2).

**Fig. 3:**
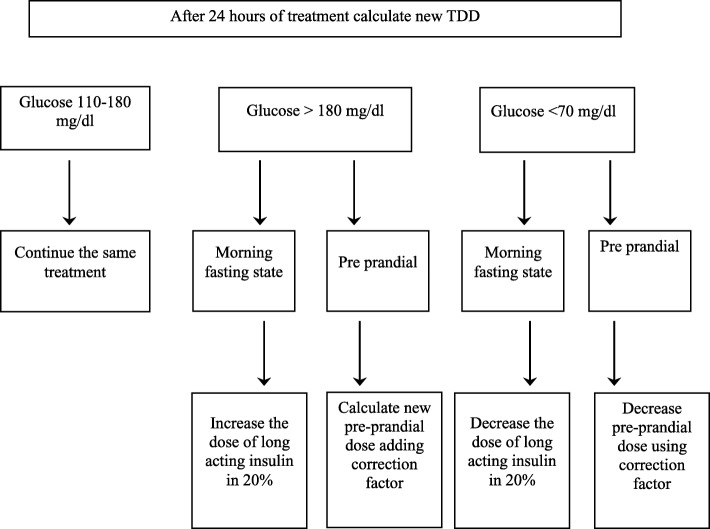
Basal –bolus protocol 24 h after admission

In accordance with our standard practice, patients with two consecutive glucose measurements above 180 mg/dl (10 mmol/l) were started on a basal–bolus insulin regimen. All oral hypoglycemic and non-insulin injectable diabetes medications were discontinued in patients eligible for insulin treatment. A total daily dose (TDD) of insulin was estimated as 0.5 units /kg/day for glucose levels of 180–400 mg/dl (10–22.2 mmol/l) on admission. Patients treated with glucocorticoids or having a glucose level > 400 mg/dl (22.2 mmol/l) were treated with 0.7 units/kg/day of insulin. The TDD was distributed as follows: 50% long-acting, defined as basal insulin, and 50% prandial fast, defined as bolus insulin. The long-acting insulins used in the study were insulin glargine (Lantus) and insulin detemir (Levemir) given in the evening. The short-acting insulins were glulisine (Apidra) and aspart (NovoRapid). We analyzed blood glucose levels by type of long-acting insulin in both patient groups (steroid-treated and control). If pre-meal glucose levels were > 180 mg/dl(10 mmol/l), a correction factor insulin was given (Fig. 2). Patients treated with insulin before admission continued their insulin regimen if blood glucose levels on admission were **<** 180 mg/dl (10 mmol/l). If more than two subsequent glucose measurements were > 180 mg/dl (10 mmol/l), correction doses of insulin were added at meal times. After the first 24 h of treatment, the insulin doses (including the additional insulin given at meal-time during the previous day) were calculated. Basal insulin doses were adjusted if the 08.00 AM blood glucose level was outside the target range (110–180 mg/dl; 6.1–10 mmol/l) for hyperglycemia as well as hypoglycemia (Fig. 3). Attending physicians were provided with the titration schedule as written in the standard protocol of care in an attempt to achieve glucose readings within the target range.

The primary endpoint of the study was to determine the differences in glycemic control between the treatment groups as measured by the mean daily blood glucose concentration. Mean blood glucose during hospitalization, daily blood glucose according to hours of point-of-care capillary blood glucose, and the number of hypoglycemic and hyperglycemic episodes were also analyzed. Additional endpoints were the number of consecutive blood glucose readings > 200 mg/dl (11.1 mmol/l) during hospitalization: patients with three or more such readings were considered not controlled and the percentage of such patients in each group was recorded.

We compared blood glucose levels of patients treated with glucocorticoids before their hospitalization to those of patients who were not treated with glucocorticoids and performed additional analysis of these two groups of patients. In the group of patients treated with glucocorticoids, we also compared blood glucose levels of patients treated with prednisone with those of patients treated with other steroids.

### Statistical analysis

We hypothesized that the difference in mean blood glucose between patients treated with steroids and those in the control group (patient with diabetes who were not treated with glucocorticoids) would be at least 20 mg/dl (1.1 mmol/l) (SD 40). A sample size of 64 patients in each study group was required for a power of 80% and alpha 5% (two sided test). The final sample included 61 patients treated with steroids and 89 patients in the control group. As a result the study power increased to 85%. Categorical variables were presented with frequencies and percent, and continuous variables were presented with a mean ± standard deviation (SD) and median. The association between the glucocorticoid-treated and the control groups and categorical variables was examined by the Chi-square test or Fisher’s exact test. Continuous variables were analyzed by T-test or Wilcoxon two sample tests. The difference in insulin dosage between the first and last day of hospitalization was calculated in each group and analyzed by the Wilcoxon signed-rank test. The statistical analyses were performed with the SAS 9.4 software. A *p* value < 0.05 was considered significant.

## Results

The demographic characteristics of the treated and untreated groups were similar, but a trend towards female sex predominance was found in the glucocorticoid group (Table 1). The mean length of hospitalization, inflammatory markers, diabetes treatment prior to admission and HbA1C levels (8.5 ± 1.9% vs. 8.6 ± 1.8%, *p* = 0.5), were the same in both groups. Treated patients received either prednisone (81%), methylprednisolone (5.9%), hydrocortisone (11%) or dexamethasone (2.2%).

**Table 1:** Clinical characteristics at admission in the glucocorticoid treated and control groups

	Glucocorticoids treated group (*n* = 61) mean ± SD	Control group (*n* = 89) mean ± SD	*p*-value
HbA1C (%)	8.6 ± 1.8	8.5 ± 1.9	0.7
Male sex (n (%))	27 (40.3%)	54(60.8%)	0.05
Age (yrs.)	70.1 ± 11.9	68.3 ± 12.1	0.4
BMI (kg/m^2^)	29.5 ± 5.2	29.6 ± 7.1	0.9
CRP^1^ (mg/L)	139.2 ± 94	136.4 ± 99.2	0.7
Prior treatment			
-Oral hypoglycemic only (n (%))	46(51.7%)	32(52.4%)	0.9
-Oral hypoglycemic and/or insulin (n (%))	68(76.4%)	43(70.5%)	0.4
Hospitalization length (Days)	16.8 ± 15.9	15.34 ± 16	0.99

The mean daily glucocorticoid dose was 42.0 ± 29.4 mg of prednisone or its equivalent. The main reason for glucocorticoid treatment was pulmonary diseases. (Table 2).

**Table 2:** Underlying conditions in the glucocorticoid group

Pulmonary	Rheumatology	Gastro	Other
COPD (14)	Still’s disease (2)	IBD (4)	Acute pericarditis (1)
Pneumonia (18)	Polymyalgia rheumatica (1)		West- Nile Encephalitis (1)
Interstitial lung (2)	Giant cell arteritis (2)		Multiple sclerosis (2)
Lung carcinoma (2)	Behcet’s disease (1)		Demyelinating disease (1)
	Gout (1)		Ig-a Nephropathy (1)
			Severe sepsis (8)

In parentheses () is the number of patients

The mean blood glucose level was significantly higher in patients treated with glucocorticoids compared with those untreated: 225 ± 48.0 mg/dl (12.5 ± 2.7 mmol/l) vs. 196.5 ± 43.1 mg/dl (10.9 mmol/l ± 2.4 mmol/l), respectively, *p* < 0.001. The mean blood glucose level at 5 PM in the glucocorticoid-treated group was significantly higher than in the untreated group: 235.8 ± 61.9 mg/dl (13.1 ± 3.4 mmol/l) vs. 183.5 ± 54.2 mg/dl (10.2 ± 3.0 mmol/l), respectively, *p* < 0.001. Similarly, the mean blood glucose level at 8 PM was also significantly higher in the glucocorticoid-treated group compared with the untreated group: 251.2 ± 74.6 mg/dl (13.9 ± 4.1 mmol/l) vs. 198.3 ± 55 (11 ± 3.1), respectively, *p* < 0.001 (Table 3). Fasting blood glucose levels and blood glucose levels measured at 12:00 noon did not differ between the two groups. Low blood glucose levels were rare during the study. Five clinically significant events of hypoglycemia (glucose values < 54 mg/dl, [< 3 mmol/l]) occurred in the study population: two in the glucocorticoid treated group and three in the untreated group. Basal and prandial insulin dosages were not significantly different between the two groups during hospitalization (*p* > 0.1). Comparing the daily insulin dose between the first and last day of hospitalization, a significantly higher daily insulin dose was recorded at the end of hospitalization only in the steroid treated group (Table 4). Nineteen patients in the steroid treated group were treated with glucocorticoids before hospitalization. Mean pre-admission steroid dose was 10 ± 7.5 mg prednisone. Mean blood glucose levels during hospitalization in these 19 patients did not differ significantly compared with the remaining patients in the group who did not receive glucocorticoids before admission: 228 ± 48 mg/dl (12.7 ± 2.7 mmol/l) vs. 212 ± 50.7 (11.8 ± 2.8), *p* = 0.3. There was no difference in mean blood glucose levels among patients in the glucocorticoid-treated group, regardless of the type of steroid they received during hospitalization. Similarly, in both study groups, the type of long-acting insulin used did not affect mean blood glucose levels.

**Table 3:** Blood glucose levels and insulin doses at 8 A.M., 12 noon, 5 P.M. and 8 P.M.

Hours	8 A.M.		12:00		5 P.M.		8 P.M.	
	Glucocorticoids	Control	Glucocorticoids	Control	Glucocorticoids	Control	Glucocorticoids	Control
BGL(mg/dl) mean ± SD	197.1 ± 52.8	184.8 ± 53.9	220.1 ± 61.1	212.9 ± 54.7	235.8 ± 61.9	183.5 ± 54.2	251.2 ± 74.6	198.3 ± 55.7
BGL mmol/l ± SD	10.9 ± 2.4	10.3 ± 3	12.2 ± 3.4	11.8 ± 3	13.1 ± 3.4	10.2 ± 3	13.9 ± 4.1	11 ± 3.1
*p*-value	0.16		0.45		<.0001		<.0001	
Insulin (units) mean ± SD	9.7 ± 3.9	9.86 ± 4.08	10.1 ± 4.3	10.2 ± 4.0	10.48 ± 3.9	9.9 ± 3.9	10.8 ± 5.4	18.7 ± 8.4
*p*-value	0.8		0.85		0.41		<.0001

**Table 4:** Difference in dosage of insulin in first and last day of hospitalization in each the groups

	Insulin dose(units) mean ± SD in first day of hospitalization	Insulin dose(units) mean ± SD in last day of hospitalization	*P*-value
Steroid treated patients	39.3 ± 19.8 [36]	46.8 ± 21.7 [44]	0.0098
Untreated patients	42.5 ± 18.1 [40]	43.9 ± 18.9 [40]	0.13

A significantly lower proportion of patients in the steroid treatment group were controlled with the basal-bolus treatment protocol, i.e. had less than three consecutive measurements of blood glucose > 200 mg/dl (11.1 mmol/l) (36% of patients in the steroid group vs. 61% in the control group, *p* = 0.003).

## Discussion

We conducted a real-life study to assess the efficacy of glycemic control achieved with a basal-bolus insulin protocol in hospitalized patients with T2DM treated with glucocorticoids compared with patients who were not treated with glucocorticoids.

Many of the patients hospitalized in internal medicine wards have inflammatory or infectious diseases and elevated markers of tissue injury such as CRP. Previous studies have suggested that serum high sensitivity (hs)-CRP levels are higher in patients with T2DM with macrovascular complications [16]. A recent study has shown that patients with higher level of hs-CRP and high HbA1c levels have higher glycemic excursion [17]. Considering that patients treated with glucocorticoids have higher levels of inflammation and thus elevated CRP levels compared to patients who are not treated with glucocorticoids, we used elevated CRP levels as an inclusion criterion for patient analysis in this study as it allowed us to maintain patient homogeneity between two groups.

Approximately two-thirds of steroid-treated patients had inadequate glycemic control compared with only one-third of the control group patients, according to the criteria arbitrarily defined for the present study. Specifically, the glucocorticoid-treated patients had higher blood glucose measurements in the afternoon and evening compared with the untreated patients. Insulin prandial doses were similar in the two groups. These findings are in agreement with those of Donihi et al. [12] who reported that excessive blood glucose levels occurred in two-thirds of hospitalized patients treated with steroids, and with those of Burt et al. [10] who found that patients treated with high-dose glucocorticoids had higher postprandial, afternoon and evening blood glucose levels and relatively mild elevations of morning glucose levels. Our findings are also in accordance with a recent study which found that the current basal-bolus insulin regimens were inadequate for controlling hyperglycemia in inpatients receiving prednisolone ≥10 mg/d [11].

Comparing the results obtained using the basal-bolus regimen with those of other reported treatment modalities suggest that the basal-bolus regimen is more effective for glycemic control. The regimen demonstrated better glycemic control and lower frequency of hospital complications compared to the sliding scale insulin regimen (SSI) for treatment of inpatients with diabetes, without increasing the number of severe hypoglycemic events [18, 19]. Gosmanov et al. [13] also found that the basal-bolus regimen is more effective than the SSI regimen for treating steroid-induced hyperglycemia, although the difference in insulin doses between the two groups (49 ± 29 units/day in the SSI group and 122 ± 39 units/day in the basal-bolus group) may have affected the results of their study. Two other studies [14, 20] compared the neutral protamine Hagedorn (NPH) insulin to glargine, both in a basal-bolus protocol, for treatment of steroid-induced hyperglycemia in hospitalized patients. Both agents were equally as effective as basal insulin for the management of these patients, and both groups experienced a similar number of hypoglycemic episodes. Ruiz de Adana et al. [14] reported that patients in the glargine group spent 42% of their hospital stay within a glucose level target range compared to 38% in the NPH group.

In a recent randomized parallel-arm study comparing two different protocols for glucocorticoid-treated patients, only 54% of patients in the basal-bolus group had blood glucose levels within the target range compared to 62% of patients in the NPH group [15]. In our study 61% of patients in the control group versus 36% in glucocorticoid treated group were adequately controlled during hospitalization. It’s clear that in the control group the main reason for insufficient control was inappropriate dose adjustment, but in the steroid treatment group more adjustments were performed during hospitalization however fewer patients achieved appropriate blood glucose control .

Considering that prednisone induced hyperglycemia takes place in the afternoon and evening, it seems reasonable to switch basal insulin glargine to insulin NPH and perform appropriate adjustments to short acting insulins. Nonetheless, several previous studies [14, 15, 20] failed to show significant improvement in glucose control using insulin NPH.

**Fig. 4:**
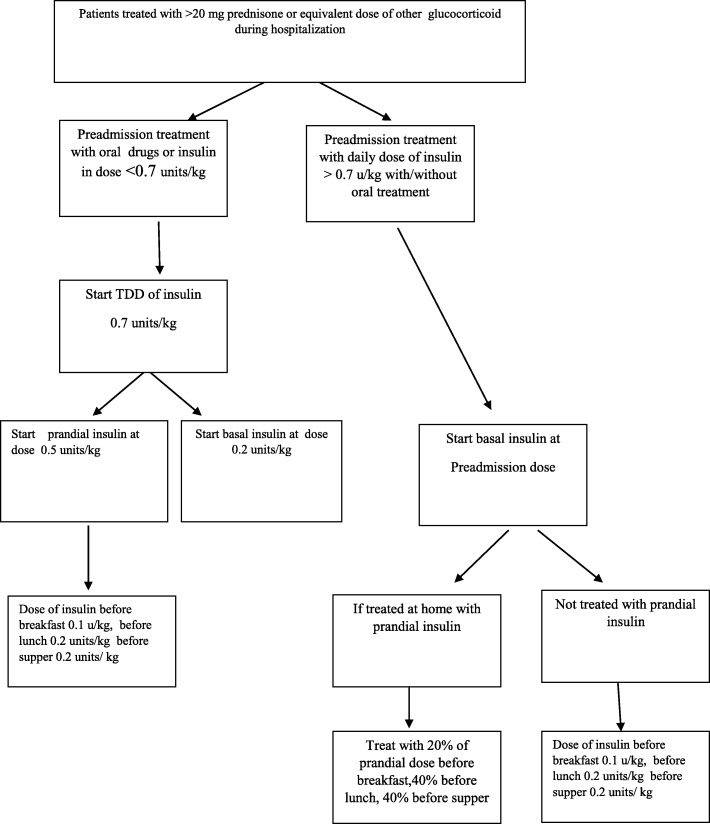
Suggested protocol for glucocorticoid treated patients

Although a significant proportion of patients hospitalized in internal medicine department are treated with steroids other than from prednisone, with different glycemic profiles, our results showed that glycemic control was not influenced by the type of steroid administered. Therefore, we think it wiser to treat all of these patients with a uniform protocol. In addition, we need to take into account that a significant proportion of patients with diabetes are treated with basal insulin on a permanent basis. In Israel insulin NPH is not used as basal insulin, so it seemed unwise to change their home insulin to insulin NPH without achieving a clear benefit in diabetes control. Patients treated with glucocorticoids have a distinct pattern of blood glucose distribution during the day (with elevation of blood glucose in the latter part of the day). This is in accordance with Burt’s study [11] which found that blood glucose control was inadequate despite of higher insulin doses in glucocorticoid treated patients. Although we were not able to address this issue in this study we speculate that a different insulin protocol would be more effective in controlling glucocorticoid treated patients during hospitalization. The protocol we suggest includes less basal insulin and a higher ratio of prandial insulin at lunch and at supper (Fig. 4).

### Study limitations

We conducted a retrospective real data study in order to evaluate the existing basal-bolus protocol in our institution and to assess its implementation. In addition to the inherent limitations due to its retrospective design, the main limitation of our study is in the failure of our patients with diabetes on glucocorticoid treatment to achieve adequate glycemic control on the basal-bolus insulin protocol. A high proportion of patients in both groups were undertreated, according to the existing protocol. We attribute this to the inadequate insulin dosing adjustments done by junior doctors and nurses responsible for the treatment of each specific patient; thus, a substantial proportion of patients did not receive an appropriate insulin dose, essentially due to excessive caution taken to avoid hypoglycemia and inability to make appropriate fine tuning, especially of prandial dosing of insulin. On the other hand, the strength of our study lies in the collecting of data about real-world patient experience thus assisting in filling the knowledge gap between clinical trials and actual clinical practice, by adding to the understanding of how best to incorporate new therapies into everyday clinical practice. We hope that our study will help to guide changes in protocols to achieve better glycemic control for inpatients receiving glucocorticoid treatment. Moreover, that this has the potential to improve the quality and delivery of medical care, reduce overall costs and improve outcomes.

## Conclusion

Treatment of hyperglycemia in inpatients with T2DM receiving glucocorticoids remains challenging. Raising the awareness of medical staff about the appropriate use of insulin protocols during hospitalization and increasing the doses of short acting insulin in the afternoon and evening in glucocorticoid-treated patients with diabetes could optimize the inherent benefits of the basal-bolus protocol.

## Abbreviations

CRP: C-reactive protein
HbA1C: Glycosylated hemoglobin A1C
NPH: Neutral protamine Hagedorn
SSI: Sliding scale insulin therapy
T2DM: Type 2 diabetes mellitus
TDD: Total daily dose
